# Hospital and Institutional News

**Published:** 1920-12-04

**Authors:** 


### HOSPITAL AND INSTITUTIONAL NEWS.

### A REPRIEVE.

We congratulate the Minister of Health on accepting the counsels of the many friends, old and new, of the voluntary hospital system who have come forward during the consideration of the Miscellaneous Powers Bill and have advocated further and careful consideration before the panic legislation at first proposed. Nobody cared much for the permissive powers that it was planned should be given to local authorities to subscribe to hospitals. They so closely resembled a general system of rate-aid that they would probably have caused a large surcease of voluntary support and at the same time did not guarantee any particular volume of financial support, the result being that the system might fall between two stools and possibly become so badly crippled as to be beyond patching up on the old lines. For all that has been said and written, few know or can guess what really is the exact hospital financial position at the moment, and Dr. Addison has been well advised in deciding to appoint an independent and impartial committee of inquiry of five suitable people who are not medical practitioners or otherwise identified with hospital management. We had hoped to announce the names of these members in the current number, but apparently the Minister has not yet completed the list. Thus that part of Clause 11 which refers to voluntary hospitals has been abandoned, and the fate of these institutions, if it has to be affected by legislation, will not be interfered with by Parliament until next year. Respecting the part of the same clause dealing with Poor-law institutions, Dr. Addison announced that a second and larger committee is to consider suggestions as to how the several thousand beds in Poor-law hospitals at present unused can economically and usefully be brought into a general health scheme.

### NATIONAL RELIEF FUND HOSPITAL GRANT.

We are informed that the Executive Committee of the National Relief Fund have appointed a special committee to advise them as to the distribution of the English and Welsh share of the grant voted towards the reduction of the war deficits of the voluntary hospitals of the United Kingdom. The total grant is £700,000, and the amount applicable to England and Wales is £560,000. The Distribution Committee consists of Sir George Murray (Chairman), Sir Napier Burnett, Rev. G. B. Cronshaw, Miss Mary Macarthur, Sir Arthur Robinson, and Viscount Sandhurst. Since the amount available will not be sufficient to allow of all deficits being paid off in full, the Committee of the Fund have decided that assistance must in general be limited to hospitals having a resident medical staff and at least fifty beds. In special cases, however, the Distribution Committee have a discretion to recommend grants to hospitals with less than fifty beds. No grant will be made in cases where the deficit for the five years ending December 31, 1919, is less than £500; and cottage hospitals, convalescent homes, and sanatoria are definitely excluded. Application forms have now been issued to all hospitals eligible for assistance.

### BRITISH HOSPITALS' ASSOCIATION.

As we go to press a meeting is being held in the Hall of the Royal Society of Medicine, Wimpole Street, convened by the British Hospitals' Association, with the object of creating a strong elective body to speak on behalf of the voluntary hospitals and to deal with the Government and Ministries in the name and on behalf of hospitals generally. The Association considers that the problems confronting London hospitals are somewhat different from those of hospitals throughout the country, and have invited representatives of only Metropolitan institutions to the conference. We hope to furnish a full report of the proceedings in our next issue. Meanwhile we would call attention to Lord Knutsford's letter on page 227.

### THE RED CROSS AND THE CENOTAPH.

Sir Arthur Stanley and Mr. Evelyn Cecil attended the Cenotaph recently on behalf of the British Red Cross Society and the Order of St. John. They deposited a wreath bearing the following inscription: " To the honoured memory of the gallant men and women of the British Red Cross Society and Order of St. John of Jerusalem in England who gave their lives for their King and Country. *Inter Arma Caritas, Pro Utilitate Hominum.*" Lady Ampthill, at the same time, left a wreath inscribed: " In proud and grateful memory of all V.A.D. members " of the same Society and Order who laid down their lives in the service of their country.

### THE PRINCE OF WALES AND TOTTENHAM.

In remarkable contrast to the hesitations of other hospitals is the decision of the Prince of Wales' General Hospital, Tottenham, to appeal for £250,000 to extend the institution. Even though the fund is associated with the Prince of Wales, and is to commemorate the successful completion of his recent tour through the Dominions, the task is a heavy one at the present time. We understand that subscriptions will be sought overseas as well as at home; and the hospital can boast of freedom from debt, a vacant site adjoining its buildings, and beds equal only to one in three of the applications. The success of this Empire Fund will depend very largely on the amount of support received from the Dominions, and that again on the thoroughness with which the facts are presented to them. Lord Gladstone, the Chairman of the hospital, records the Prince's approval and support, and presumably neither would have been forthcoming unless the scheme had been carefully prepared.

### CASUAL HELP AND WEEKLY GIFTS.

The recent sally of the medical students of the London Hospital into West-End theatres and restaurants, which produced nearly £1,500, is to be repeated, we understand, at unadvertised intervals throughout the season. Whether the plan, shorn of its novelty, will produce the reaction that perpetual "Flag Days" excited remains to be seen. Its chief value, probably, is in the advertisement which it gives to the needs of the moment. Welcome as the proceeds are, even more welcome have been the cheques which followed the raid from people who had not hitherto realised the necessity and may become annual subscribers. The method can hardly become a regular or permanent source of income. It is on this that efforts should concentrate. Mr. Philip Inman has received over a hundred letters from firms anxious to subscribe weekly to hospitals since the lead lately given by Messrs. W. H. Smith & Son. That is an encouraging sign, and if the employers will do their part as well as the staffs we may yet win through this critical period.

### HOSPITAL SATURDAY IN BIRMINGHAM.

At a meeting called for the purpose of the distribution of the Birmingham Hospital Saturday collections amongst the local medical charities recently, Sir David Brooks (Chairman) referred to the misunderstanding which had unfortunately arisen in connection with the establishment by the General Hospital of a league for the benefit of that hospital alone. A good deal of feeling exists amongst other hospitals that the scope of the league should be widened so as to include all the hospitals of the city. The Chairman of the General Hospital (Mr. C. H. Saunders) expressed regret that the misunderstanding should have arisen between the General Hospital and the other hospitals, and he hoped that it would soon be removed. Without minimising the claims of the other hospitals, the General, Mr. Saunders pointed out, is in a very desperate financial position, and it was because their Board felt that some attempt must be made at once to increase its income that the General Hospital League was inaugurated. The amount collected by the Saturday Fund shows a considerable increase—namely: 1919, £43,036; 1920, £60,000. This result is mainly due to the big increase in workpeople's contributions, most of whom are now subscribing 2d. weekly to the Fund.

### THE CINEMA IN HOSPITAL PROPAGANDA.

The first portion of the scheme for the extension of the Chesterfield and North Derbyshire Royal Hospital, a new out-patient department, is now well in hand, and the foundation-stone was laid in masonic form by Mr. N. J. Hughes-Hallett, Clerk to the Derbyshire County Council and Deputy Provincial Grand Master of the Derbyshire Freemasons, on November 6, when £168 was laid on the stone by the various Lodges, and contributions from individual masons and a collection on the ground brought the total to £370. A film of this picturesque ceremony was taken, and will be exhibited at the local cinema houses, when collections will be made which, it is hoped, will add materially to the Hospital Extension Fund. This is not the first occasion on which the cinema film has been brought into use by the hospital, a film depicting an "accident" at a colliery in the neighbourhood, its progress in the ambulance on the road, arrival at the hospital, treatment in the casualty department and ward, and discharge from the institution "cured" being one of the means of advertising the needs of the hospital three years ago. A sum of £17,500 is now needed to complete the £50,000 necessary to carry out the extension scheme.

### PATIENTS' PAYMENTS DAY BY DAY.

At the annual court of Governors of the Royal Chest Hospital, City Road, Mr. Stuart de la Rue said that it went against the grain to think about payments by patients, but that they would have to be introduced in the near future. His own scheme was to persuade the community during good health to make contributions to the hospitals in the form of insurance against sickness, and thus become entitled to attendance. Mr. L. B. Keyser, the treasurer of the Royal Hampshire County Hospital, at the recent court of Governors, deplored Lord Knutsford's S.O.S. to the Government. In Hampshire, he stated, they have adopted a scheme of payment by patients and weekly contributions from workmen. The secretary of the Royal Westminster Ophthalmic Hospital informs us that the committee have decided to charge in-patients according to means, but in no case above the cost of maintenance as shown in the statistical tables of the annual report. An almoner has been appointed to assess the amount to be charged.

### REDRUTH HOSPITAL EXTENSIONS.

The building scheme for Redruth Miners' and Women's Hospital has now been decided in detail. The original estimate of £8,000, which was generously provided in equal shares by Sir E. Nicholl and the British Red Cross Society, has been since exceeded, so that the complete scheme has been somewhat curtailed. But a new hostel for the nurses, with central heating limited to that, further medical beds, and some structural alterations have been determined on. The postponed improvements are a complete installation of heating for the men's and women's sides, which will jointly cost £850, and the completion of the $x$-ray apparatus, to cost £250 more. Since the sum already received was promptly given from only two sources, is it too much to hope that the Committee may be able to raise the remaining £1,100 from the general body of supporters? It would be a pity not to complete the scheme now that only a final effort is required to find the money.

### A LOSS TO PHILANTHROPY.

The philanthropic world has sustained a serious loss through the death of Mr. William Baker, the Chairman of the Council of Dr. Barnardo's Homes, at the age of seventy-two years. Mr. Baker, who worked for many years with Dr. Barnardo, and became honorary director of the Homes upon the latter's death in 1905, is said to have seen some 30,000 children started in life during the fifteen years he has filled that position.

### AN INTERESTING PROPOSAL AT NORWICH.

The destination of funds for schemes which eventually prove abortive is a delicate matter, but many will sympathise with a suggestion made by Mr. Charles Larking, chairman of the Finance Committee of Norfolk and Norwich Hospital. After describing the grave financial position of the hospital, Mr. Larking referred to the ambitious local war-memorial scheme which, he said, had ended in failure. He added that the scheme, which could not claim to be, in reality, a war memorial, had diverted subscriptions from other legitimately designated schemes, including those of the Norwich hospitals. He, therefore, asked if those who had promised £60,000 to the abortive scheme could not agree to transfer their donations to one or other of the local hospital appeals. We hope that the suggestion will be considered immediately, and that a public meeting be held to discuss it.

### FROM A HOSPITAL TO LANDSCAPE GARDEN.

By an irony of circumstance the site of the old infirmary, Manchester, which became vacant when the great new hospital was built, has proved a white elephant; though it was the new institution which some thought might become so at the time when the change was made. The use to which the old site should be put is still undecided. A picture gallery, and now a Dutch garden, have been pressed unsuccessfully upon the city council. An economical improvement on the latter suggestion has since been made by a correspondent who advocates a natural garden, one, that is to say, where contours would be formed by the existing inequalities of the derelict ground. This plan, if the site is to become a garden, would probably be much cheaper than any artificial design, and need be no less beautiful. The writer suggests that the transformation might be made free of cost if the city council would allow the local nurserymen the advertisement of displaying their flowers and garden designs free for a reasonable period. If these several efforts were harmonised with one design, there is a good deal to be said for the proposal. How much longer is the disgraceful condition of the old site to remain and its numerous possibilities to be wasted?

### SITUATIONS IN LONDON.

Though London salaries are often higher than those prevailing elsewhere, it does not necessarily follow that people will "better themselves" by accepting situations under local authorities in the Metropolitan area. This is shown in a Willesden Council report. The Chairman of the Health Committee interviewed four selected candidates from the provinces for the position of sanitary inspector, but had been unable to fill the vacancy, as none of the candidates would accept the position owing to the dearth of housing accommodation, the high rents, and cost of living prevailing, and the excessive cost of removal to London. The Council has asked the Chairman to appoint an applicant already residing in or around the Metropolis.

### RESIGNATION OF A HOSPITAL COMMITTEE.

In consequence of the appointment of Dr. MacLeod to the staff of Evesham Hospital, two other members of the medical staff resigned, and patients have had to be sent to the neighbouring hospitals. The committee, therefore, called a meeting of subscribers to seek sanction for its action, but the subscribers called upon the committee to resign, which it has since done. This situation is unusual, for when a medical staff is opposed to a committee, the opinion of the hospital's supporters is rarely unanimous on the doctors' behalf.

### CO-ORDINATION AT CARDIFF.

It being reported that, while the waiting-list at King Edward VII. Hospital, Cardiff, numbers 1,057, there are vacant beds at the Prince of Wales' Orthopædic Hospital there, the emergency and reference committee of the former has been instructed to see if some co-ordination is not possible. Mr. Andrew Brown, who raised the question, declared that of the fifty-four beds in the Prince of Wales's Hospital only seventeen had been occupied for some time. He remarked that the waiting-list included many cases eligible for the treatment provided at the Prince of Wales's Hospital, which was intended to admit civilian patients when its work for ex-Service men had been accomplished. We believe that, subject to this condition, the committee of the Prince of Wales's Hospital is favourably disposed, and that Mr. Brown's suggestion will bear fruit. Another attempt at co-ordination is seen in the proposal to ask the Lord-Lieutenant of Glamorganshire to call a conference of representatives of the hospitals, convalescent homes, and Guardians in the county to discuss what can be done to reduce the waiting-list at King Edward VII. Hospital. Over the length of this list the public is growing impatient; but the fact remains that, apart from an overdraft, the hospital still requires an additional £36,000 a year. A conference of representatives of all grades of Labour is to be called to consider how to increase the hospital's revenue. Some trade unions still do not contribute.

### THE EDINBURGH MEDICAL MISSIONARY SOCIETY.

There is one branch of the medical profession which is not overcrowded, and that is the branch of medical missions. In some interesting statistics the Edinburgh Medical Missionary Society states that of the 45,000 doctors on the British Register 400 only are medical missionaries. The Society itself, which was founded in 1840, has at the present moment vacancies for eighty men and women. It maintains two hospitals at Nazareth and Damascus, beside the Cowgate Dispensary in Edinburgh. At the latter practical training is given, both in professional and in religious work. Practically all the officers of the Society are medical men, and they wish to bring the Society's work before their professional colleagues. Besides supplying fully qualified practitioners to all the Churches requiring medical missionaries, the Society gives an opportunity to those who seek useful work in an interesting field of medicine which is perhaps alone in being unstocked. The interest of the work, the travel which it occasions, are compensations for the limited prospects. If medical missionaries do not achieve a fashionable practice, they are spared the competition and chances of failure incidental thereto. Their cases, their patients, and their centres of work are of great interest, and it must only be because this branch of medicine is little known that more students do not join it. Dr. Lechmere Taylor is the secretary of the Society, whose office is at 56 George Street, Edinburgh.

### THE HOSPITAL INSURANCE SCHEME.

Following Mr. Coles's suggestion of adding institutional benefit to the National Health Scheme, through a small extra weekly premium, Major George Hamilton, M.P., brings forward a development of the idea. He thinks, in accordance with the policy we have already suggested, that the most appropriate State service that could be rendered to the hospitals is by the logical extension of the insurance system. A weekly penny charge would yield a pool of four or five million sterling, which could be drawn upon by the hospitals according to services rendered. The faulty part of Major Hamilton's idea lies in the connection between the panel doctor and the hospital. To put it shortly, we understand that it is suggested that the panel doctor should send on his patient with a card saying what is required—an operation or whatever else appears necessary. Then this card would be cashed with the pool at its face value, presumably according to a scale regulated on services given. Possibly quite inadvertently Major Hamilton suggests something like dictation on the part of the panel practitioners, which, of course, would not do. But in any case, the suggestion has excellent points which could easily be worked out by somebody who knows what the inside of a hospital is like.

### HEALTH IN UNDERGROUND CAFES.

Is the work in underground tea-shops injurious to the waitresses? No one in London seems to have bothered about this matter. In Sydney, New South Wales, it appears, allegations have been made that the health of women was affected through working in café basements, which, apparently, exist there as here. The municipal council asked Miss Blomfield, the lady sanitary inspector, to inquire into the subject, and she has since reported that all the women were found to be in good health, and that there was no ground for allegations to the contrary.

### HOW NOT TO EAT.

We are sorry to hear the opinion of Sir Harry Baldwin, expressed during a lecture at the Central Y.M.C.A., Aldersgate Street, that tea is a dangerous meal, because tea and bread-and-butter have a depressing influence on the production of saliva. The social and refreshing uses of this most important of the lesser meals have so greatly been valued by the majority of people that Sir Harry's hope, expressed after he had thoroughly frightened his audience, that the deleterious effect may be neutralised by placing slices of orange between those of bread-and-butter, gives some encouragement that one of the pleasures of life is only modified, not lost. To go on as we have been going on, with this fresh and horrid knowledge leading our thoughts saliva-wards during tea-time, would be too awful! Sir Harry says that one great cause of irregularities in the growth of teeth was the contracted jaw brought about by breathing through the mouth. Through keeping it open the pressure necessary to shaping the jaws is eliminated. The aim should be to promote saliva, which is a natural mouth-wash, and he advises the eating of fruits and salads at the end of every meal.

### THE LYING-IN PERIOD.

The attention of the Health Committee of the Westminster City Council has been called to the fact that it is the practice of lying-in hospitals and homes to discharge women confined in such institutions ten days after the date of their confinement. The Committee says that, from information which has come to its knowledge, it has formed the opinion that women should be retained in these institutions for at least a fortnight following confinement. In many cases the Ministry of Health makes grants in aid of the expenditure of these institutions in respect of maternity services rendered by them, and in these circumstances the Health Committee proposes to get in communication with the Ministry on the matter and to ask it to consider the question of the desirability of making it in future a condition of the Government grant that mothers shall not be discharged from the institution until fourteen days have elapsed following confinement, or such further period as may be necessary in cases requiring medical treatment. The obvious difficulty is that such a provision means treating 40 per cent. fewer cases, unless large extension and maintenance schemes can be financed.

### THIS WEEK'S DRUG MARKET.

Business shows no sign of improvement, and the downward tendency of prices continues. So long as values decline buyers will restrict their purchases to immediate requirements, but, should the tone become steadier for one reason or other, buying would probably be resumed, for stocks in the hands of distributors must need replenishing in many cases. Quinine is obtainable from dealers at below makers' prices. Citric acid is very quiet, and lower. Cocaine is offered at cheaper rates, and is in good supply. Cream of tartar is lower. Hexamine, acetyl-salicylic acid, salicylic acid, sodium salicylate, phenazone, and various other synthetic drugs have a lower price tendency. A fair business has been done in opium at steady rates. Ipecacuanha is in plentiful supply, and prices have a downward tendency. Eucalyptus oil is in steady demand.

